# Therapeutic Effect of Darkling Beetle (*Zophobas morio*) Hemolymph on Skin Thermal Injury in Mice Infected by *Staphylococcus haemolyticus*

**DOI:** 10.3390/vetsci8120319

**Published:** 2021-12-10

**Authors:** Shu-Xian Li, Ning Liu, Meng-Ze Du, Yao-Hong Zhu

**Affiliations:** 1 Department of Veterinary Clinical Sciences, College of Veterinary Medicine, China Agricultural University, Beijing 100193, China; li_shuxian2021@163.com (S.-X.L.); nliu2224@163.com (N.L.); 2 Animal Science and Technology College, Beijing University of Agriculture, Beijing 102206, China

**Keywords:** *Z. morio* hemolymph, thermal injury, inflammation, *Staphylococcus haemolyticus*, mice

## Abstract

Staphylococci are the most common pathogens isolated from skin infections in livestock or companion animals. Antibiotic therapy is the best treatment for infections, but local or systemic use of antimicrobials increases the risk of bacterial resistance. Insects are rich in antimicrobial peptides, which can reduce bacterial resistance and can be used to treat bacterial infections after skin burns. We propose that the use of the darkling beetle (*Z. morio*) hemolymph to treat skin infections in mice by *Staphylococcus haemolyticus* is one of the alternatives. *Z. morio* hemolymph alleviated the increase in wound area temperature in mice with a skin infection, reduced the bacterial load of the wound, and accelerated the wound healing speed significantly. Pathological sections showed that *Z. morio* hemolymph can significantly reduce inflammatory cell infiltration, and promote skin tissue repair. Real-time fluorescent quantitative polymerase chain reaction (PCR) revealed that the *Z. morio* hemolymph can significantly reduce the levels of pro-inflammatory cytokines, including interleukin-1 beta (IL-1β), interleukin-6 (IL-6), tumor necrosis factor-alpha (TNF-α), and chemokine interleukin-8 (IL-8). Our findings suggest that *Z. morio* antibacterial hemolymph can promote wound contraction, relieve local inflammatory responses and promote wound healing in mice infected with a heat injury, which has a positive therapeutic effect and enormous potential for skin thermal injury.

## 1. Introduction

Staphylococci are a group of bacteria that play an important role in clinical, veterinary, economic and agricultural fields, as they have strong resistance to antimicrobials and various virulence factors [1]. Domestic pets are considered potential reservoirs of zoonotic pathogens [2]. *Staphylococcus aureus* can be isolated from the skin or mucosa of 67.3% dogs and 73.8% cats in 383 registered companion pets [3]. Infection of methicillin-resistant *Staphylococcus* was reported in the skin and soft tissues of the horse, and it can be colonized in clinical personnel [4,5]. Bovines are the most susceptible hosts to *Staphylococcus* infection [6]. Many coagulase-negative and -positive staphylococcal species can be isolated from goat skin infections and secondary to other primary skin diseases [7]. Bacterial infection disrupts the wound repair process, resulting in delayed wound healing, aggravating the infection. *S. haemolyticus* is one of coagulase-negative *Staphylococcus* (CoNS) isolated from infected human clinical samples [8]. It is involved in opportunistic infections in immunocompromised patients, leading to an increase in the prevalence of hospitalized patients [9].

Skin wound healing is a dynamic and complex process, which includes several stages, such as inflammation, proliferation, granulation tissue formation and tissue remodeling [10]. Cytokines are important mediators in the inflammatory response after burning [11]. Early inflammatory markers, include interleukin-1 beta (IL-1β), interleukin-6 (IL-6), tumor necrosis factor alpha (TNF-α), and chemokine interleukin-8 (IL-8) [12]. A previous study reported that the levels of IL-1β, IL-6 and TNF-α were closely related to organ failure in burned mice [13].

Due to the antimicrobial resistance among bacteria, conventional therapy is not ideal for burns wound healing. Antimicrobial peptides (AMPs) have become a new class of effective drugs for treating skin trauma because of their own characteristics and the advantages of reducing bacterial resistance [14]. AMPs accumulate on the surface of infected skin and mucosa, regulate the immune system, and promote every step of skin wound healing [15]. A novel antimicrobial photodynamic peptide AMP2-Ce6 was reported to accelerate wound healing in mice infected with *S. aureus* [16]. Our previous study showed that the antibacterial protein extracted from the darkling beetle (*Zophobas* *morio*) had a high safety, stability, and antibacterial effect on the pathogen of bovine mastitis, including *S. aureus*. An in vitro study proved that the *Z. morio* hemolymph can reduce the expression of pro-inflammatory factors, including interleukin-1β (IL-1β) and interleukin-18 (IL-18) [17], which suggests that *Z. morio* hemolymph can be used as a novel therapeutic drug to reduce pathogen infection in animals. Nevertheless, we hypothesize that *Z. morio* hemolymph in vivo to treat *S. haemolyticus* infection, remains largely understudied.

In this study, we successfully established a mouse skin thermal injury model infected by *S. haemolyticus* to evaluate the therapeutic effect of *Z. morio* hemolymph for treatment on heat-damaged skin wounds, providing data support for further development of the utilization value of *Z. morio* hemolymph.

## 2. Materials and Methods

### 2.1. Ethics Statement

The breeding and experimental operations of all experimental animals were carried out in compliance with the Guidelines for Laboratory Animal Use and Care from the Chinese Center for Disease Control and Prevention and the Rules for Medical Laboratory Animals from the Chinese Ministry of Health, under protocol AW51701202-2-2, approved by the Animal Ethics Committee of China Agricultural University. All operations were performed using Zoletil (WK001, Virbac, France) to minimize the patient’s pain.

### 2.2. Animals and Experimental Design

All studies were conducted in Kunming mice aged 6–8 weeks, (weight of 24–29 g). These mice were raised in the animal experimental facility of the College of Veterinary Medicine, China Agricultural University, Beijing, China. The mice were kept in community cages (5–10 mice per cage) and provided free access to drink fresh water and eat mouse chow. These mice were randomly divided into four groups, receiving a different treatment, (1) control group: no thermal injury and infection operation; (2) saline group: thermal injury on day 0 and challenge with *S.* *haemolyticus* No.11478 1 × 10^8^ colony-forming unit (CFU), saline was used to smear the wound, two times a day; (3) erythromycin group: thermal injury and inoculation with *S. haemolyticus* No.11478 (1 × 10^8^ CFU), the wound was smeared with erythromycin ointment, two times a day and (4) XTRC beetle group: thermal injury and inoculation with *S. haemolyticus* No.11478 (1 × 10^8^ CFU), the wound was smeared with *Z. morio* hemolymph, two times a day. Bacterial inoculation was carried out only on day 0. To ensure the success of modeling, bacteria inoculation was performed twice (each interval of 4 h). *Z. morio* hemolymph or erythromycin was applied to the thermally injured skin area of mice for the first time at 4 h after the second inoculation. Subsequently, only medication was performed every day, and no bacteria were inoculated. The dosage of hemolymph antibacterial protein or erythromycin was 5 mg.

### 2.3. Bacterial Strains and Drugs

*S. haemolyticus* NO.11478 was isolated from the bovine skin wounds. Erythromycin ointment was purchased from Mayinglong Pharmaceutical Group Co., Ltd., Dingzhou, Baoding, China. The extraction and preparation of the antibacterial hemolymph of *Z. morio* were carried out according to the preliminary research results of our laboratory [17].

In this study, preparation chromatography was used to purify the hemolymph of *Z. morio*, and the effective antibacterial components in the hemolymph of *Z. morio* were analyzed by shotgun proteomics. *Z. morio* hemolymph contained coleoptericin, attacin, and other components.

### 2.4. Mouse Infection Model

The fur on the back of each of the mouse was removed by an electric hair clipper, and the fine hair was shaved off with a razor. The equipment for simulating thermal injury is a self-fabricated temperature-controlled simulator. We created a full-thickness thermal injury on the back of each mouse. The shape of the soldering iron tip is a square with a side length of 6 mm, the soldering iron temperature was set to 150 °C. Zoletil was used to anaesthetize mice by intraperitoneal injection. Then, the iron tip of the thermal injury simulator was used to touch the hairless skin for 6 s to form a thermally injured area. After the thermal injury, 1 × 10^8^ CFU in 10 uL phosphate-buffered saline (PBS) (Solarbio, Beijing, China) of *S. haemolyticus* NO.11478 was inoculated on the thermally injured surface, wrapped with sterile gauze and fixed with pressure-sensitive tape. In order to ensure the success of the infection model, the infection process was repeated after 4 h.

### 2.5. The Temperature of Wound Area and Weight Measurement

During the experiment, the wound area temperature of all mice was monitored with an infrared thermometer (Bestone, Shenzhen, China) from the first day. The weight of mice was measured every other day from the first day.

### 2.6. Peripheral Blood White Blood Cell Count

On days 3, 5, and 7 after infection, blood was collected from the orbit of the mice. The fresh blood was enriched in a collection tube containing EDTA-K2, and then the blood was sent to the Laboratory Department of the Animal Hospital of China Agricultural University (Beijing, China) to detect and count the number of peripheral blood leukocytes.

### 2.7. Bacterial Examination of Wound Skin Biopsy

On days 3 and 5 post-infection, the mice were euthanized, and the skin was collected for bacterial count. The infected skin tissue was placed in a small centrifuge tube (Bioeasy, Beijing, China). Sterilized saline and small steel balls were added and placed in a tissue homogenizer (Scientz, Ningbo, China) at 45 HZ for 90 s. The tissue was continuously diluted 10 times, and the liquid was coated on the Mueller Hinton Agar (MHA) (Aobox, Beijing, China) plate. Each group was repeated three times, and incubated at 37 °C. The plate was counted after 18 h. 

### 2.8. Wound Area Measurement

The sizes of mouse skin wounds were measured by vernier calipers (Anyi, Guilin, China) to compare wound healing speeds between different groups.

### 2.9. HE Staining of Murine Thermally Injured Epidermis and Inflammatory Cell Quantification

As previously described [18], thermally injured skin specimens were fixed in 4% paraformaldehyde (Solarbio, Beijing, China). After the thermal injury, epidermis samples were dehydrated in a series of ethanol gradients and embedded in paraffin, The samples were stained with hematoxylin-eosin (HE) (Beyotime, Beijing, China) and observed under an optical microscope (Olympus, Tokyo, Japan). The number of inflammatory cells infiltrating was identified and counted on every 0.2 mm^2^ area at regions between epidermis and dermis on HE-stained section with an optical microscope (Olympus, Tokyo, Japan) [19]. The data were expressed as the mean of all areas counted ± standard deviation of the mean (SEM).

### 2.10. RNA Extraction and Quantitative Real-Time PCR

The total RNA was extracted from the normal skin and thermally injured skin using RNAiso Plus (Takara, San Jose, CA, USA). The synthesis of complementary DNA (cDNA) was performed with the PrimeScriptTM RT Reagent Kit with gDNA Eraser (Takara, San Jose, CA, USA) with gDNA Eraser for reverse transcription. The cycle threshold (CT) value of the target gene was normalized with the cycle threshold (CT) value of the housekeeping gene, β-actin as the control. The PCR primer sequences are shown in Supplementary Table S1. Relative mRNA expression results were presented as fold-change using the 2^−ΔΔCT^ method.

### 2.11. Separation of Effective Components in Z. morio Hemolymph by Liquid Chromatography

The *Z. morio* hemolymph protein was purified by the AKTA avant protein purification instrument (GE Healthcare, Fairfifield, MA, USA). GE Superdex 100 pg Preloading column filler was used for preparative chromatography. The liquid volume of the sample protein was 2 mL, and the concentration of the sample protein was 50 mg/mL. The column temperature was maintained at 25 °C, and the UV absorbance was monitored at 280 nm. The flow rate was 2 mL/min, and the mobile phase was phosphate-buffered saline (pH = 7.2).

### 2.12. Shotgun Proteomic Analysis of Antibacterial Protein Components in Z. morio Hemolymph

A shotgun proteomic analysis was performed on the uninduced *Z. morio* hemolymph, on the antibacterial protein solution after ultrafiltration, and the antibacterial protein components of *Z. morio* hemolymph, which was purified by a molecular sieve mentioned in 2.11. The protein cleavage was used by SDT (4% SDS, 100 mM Tris-HCl, 1 mM dithiothreitol (DTT), pH 7.6), and the quantification was measured by Beyotime Biotechnology BCA kit (Shanghai, China). The protein sample was digested with filter-aided sample preparation (FASP), and the peptide was desalted by C18 Cartridge (66872-U Sigma, Shanghai, China. After lyophilizing the peptide, 40 μL 0.1% formic acid solution (FA, 06450 Fluka, Charlotte, NC, USA) was added to redissolve, and the peptide was quantified. Each sample was separated by the HPLC system Easy nLC (Thermo Scientific, Waltham, MA, USA) and analyzed by a Q-Exactive mass spectrometer. The MaxQuant software (version 1.5.3.17, Max Planck Institute of Biochemistry, Martinsried, Germany) was used for database mass spectrometry and quantitative analysis.

### 2.13. Statistical Analysis

Experiments were conducted at least three times. Statistical analysis was performed through the GraphPad Prism7 software (version 7, GraphPad Software Inc., San Diego, CA, USA). For two-group comparisons with Gaussian distribution, a two-tailed unpaired *t*-test with Welch’s correction was applied when the variances of two groups were proved equal by the F test. For two-group comparisons with non-Gaussian distribution, a Mann–Whitney test was applied. For multigroup comparisons with Gaussian distribution, analysis of variance (ANOVA) with Tukey–Kramer’s multiple-comparison test was used after confirming the homogeneity of variance was confirmed by Bartlett’s test. For multigroup comparisons with non-Gaussian distribution, a Kruskal–Wallis test with Dunn’s test was used. A value of *p* < 0.05 was considered statistically significant.

## 3. Results

### 3.1. Main Antibacterial Components of Z. morio Hemolymph

The hemolymph antibacterial protein was filtered through the GE Superdex 100 pg chromatography column, and there were six peaks. A total of eight tube eluents were collected for in vitro antibacterial test, and two tube eluents containing four antibacterial components showed antibacterial effect (Supplementary Figure S1). Affinity liquid chromatography and mass spectrometry were used to analyze the above eluents containing antibacterial components, the uninduced hemolymph of *Z. morio* and the induced hemolymph of *Z. morio* (Supplementary Figure S2), and then compared with the database. The four main components of the hemolymph of *Z. morio* are shown in Table 1.

### 3.2. Effect of Z. morio Hemolymph on Body Weight and Wound Area Temperature of Thermally Injured and Infected Mice

On the 1st day after thermal injury, the wound area temperature of each thermally injured mouse was significantly higher than that of the control group. Three days after thermally injured, the wound area temperature of mice in the erythromycin and the hemolymph treatment groups was significantly lower than that in the saline group (Supplementary Figure S3a). The wound area temperature of mice in the *Z. morio* hemolymph group was close to that in the normal control group from the 2nd day. During the whole experiment, the body weight of each thermally injured and infected mouse increased, and the body weight of the hemolymph antibacterial protein treatment group and saline groups was higher than that of erythromycin treatment group (Supplementary Figure S3b).

### 3.3. Z. morio Hemolymph Reduces the Load of S. haemolyticus in Wound Tissue and Leukocytes Count in Peripheral Blood of Thermally Injured Mice

**Figure 1:**
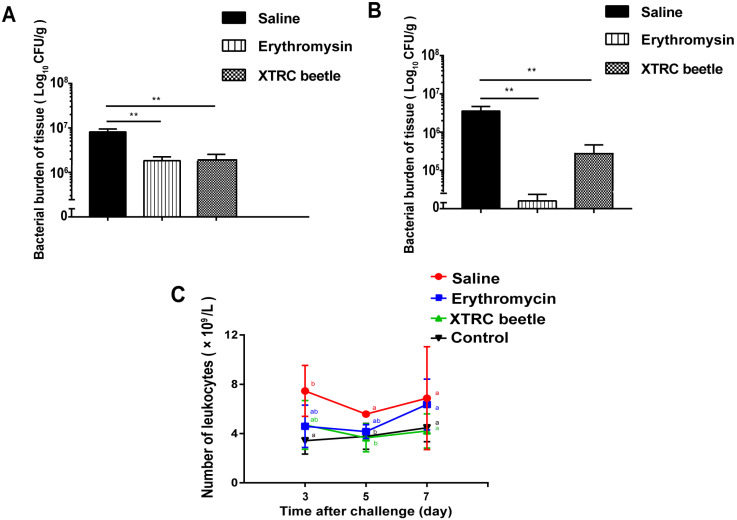
Effects of different treatments on bacterial load and peripheral blood leukocytes count in thermally injured and infected mice. (**A**) Bacterial load on wound of mice on day 3. (**B**) Bacterial load on the wound of mice on day 5. ** *p* < 0.01. (**C**) Results of leukocytes count in peripheral blood of mice on days 3, 5, and 7 after thermally injured and infection. Mean values at the same time point without a common superscript (a,b) differ significantly (*p* < 0.05; Tukey’s test). The data are expressed as the mean ± standard error of the mean (SEM), *n* = 6.

On the 3rd and 5th days after thermal injury, the number of wound bacteria in the erythromycin and the hemolymph antibacterial protein treatment groups was significantly lower than that in the saline group (Figure 1A,B). Day 3 after thermal injury, the total number of leukocytes in blood routine examination was higher than that in the normal control group. There was a significant difference between saline and normal control groups. On the 5th day of blood routine examination, the total number of white blood cells in normal, control, and hemolymph treatment groups was lower than that in the saline group and there was a significant difference. On the 7th day of blood routine examination, the total number of white blood cells in the blank control, erythromycin treatment, and hemolymph antibacterial protein treatment groups was lower than that in the normal saline group (Figure 1C).

### 3.4. Z. morio Hemolymph Antibacterial Protein Promotes Wound Repair in Mice

**Figure 2:**
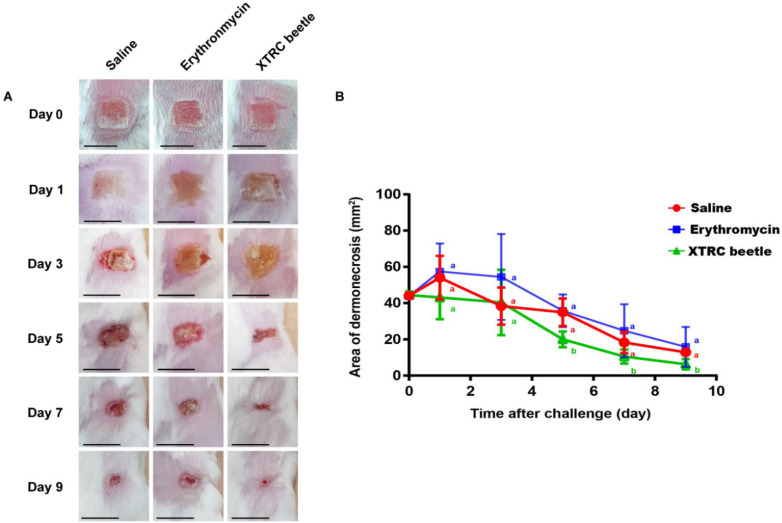
Wound healing and wound area measurement in thermally injured skin of mice treated with saline, erythromycin, and *Z. morio* hemolymph alone on days 0, 3, 5, 7, and 9. (**A**) From day 0, every other day, the wound condition of mice in each group after thermal injury and infection. Scale bars, 6 mm. (**B**) From days 0 to 9, the wound area of each group was measured with a vernier caliper every other day. Mean values at the same time point without a common superscript (a,b) differ significantly (*p* < 0.05; Tukey’s test). The data are expressed as the mean ± standard error of the mean (SEM), *n* = 6.

On days 1 and 3 after thermal injury, there was no significant difference in the wound area of mice between the saline, the erythromycin treatment, and the *Z. morio* hemolymph antibacterial protein group (Figure 2B). Nevertheless, the wound area of the *Z. morio* hemolymph antibacterial protein treatment group was smaller than that of the saline and erythromycin treatment groups on day 5. On the 5th day of infection after skin thermal injury, the re-epithelialization rate of the *Z. morio* hemolymph treatment group was 55 ± 3.56%, the erythromycin treatment group was 18 ± 13.53%, and the normal saline group was 25.49 ± 4.42%. On the 7th day of infection after skin thermal injury, the re-epithelialization rates of *Z. morio* hemolymph, erythromycin, and normal saline groups were 76 ± 7.51%, 52 ± 5.91%, and 59.48 ± 11.40%, respectively. On the 9th day of infection after skin thermal injury, the re-epithelialization rates of *Z. morio* hemolymph, erythromycin, and normal saline groups were 86 ± 5.13%, 69 ± 12.47%, and 70 ± 3.23%, respectively (Figure 2B). We could see the same results from the photos (Figure 2A). 

### 3.5. Z. morio Hemolymph Promotes Wound Healing in Mice Infected by S. haemolyticus

**Figure 3:**
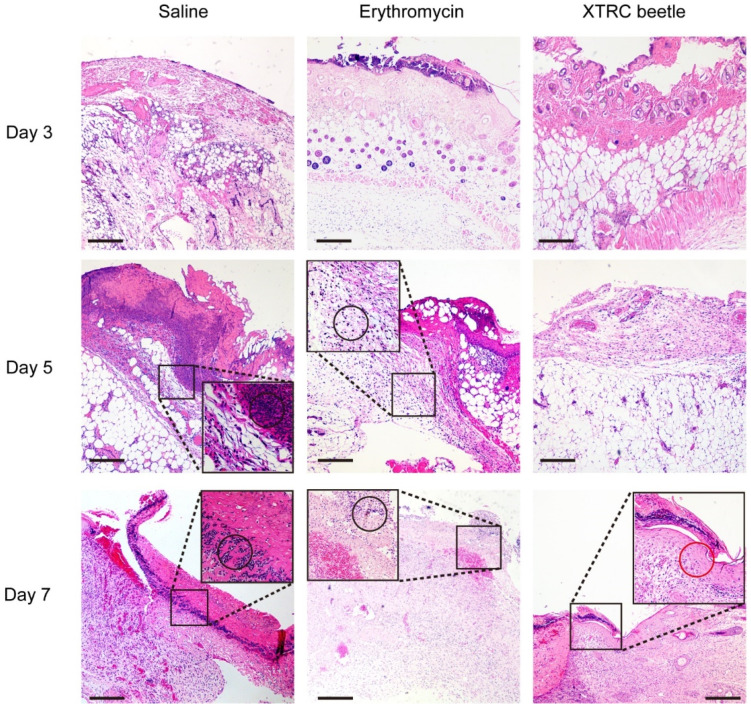
Representative images of hematoxylin and eosin (HE) stained histological sections of wound healing tissues from infected mice treated with erythromycin ointment or *Z. morio* hemolymph, and infected untreated mice on days 3, 5, and 7 after infection. The squares indicate the enlarged area, the black circles represent inflammatory cells, and the red circles represent the new epidermis. Scale bars, 300 μm, *n* = 4.

According to the pathological section of each group on day 3, the burnt epidermis of the three treatment groups was necrotic, and injury and hair follicles were damaged. Vascular dilatation was obvious in the deep dermis, and collagen fibers were arranged and filled with a large number of red blood cells and inflammatory cells (Figure 3).

From the histopathological results on day 5, the normal structure of the epidermis in the control group was unable to identify, which contains a large number of inflammatory cells and cell debris. The structure of the nipple layer could not be distinguished. The reticular layer contained a large number of inflammatory cells, and collagen fibers were swollen and necrotic. The epidermis and dermis of the erythromycin group were similar to the control group, but the number of inflammatory cells and cell debris in the epidermis was less. A large number of granulation tissue layers appeared in the epidermis of *Z. morio* hemolymph group. The dermis had edema and inflammatory cell infiltration (Figure 3).

In the control group, there were many inflammatory cells and inflammatory cell debris in the necrotic epidermis on day 7 after thermal injury. A large number of new granulation tissues were formed in the dermis, and a large number of inflammatory cells were visible. The epidermis structure of erythromycin protection group was indistinguishable, and there were a large number of new granulation tissues in the dermis. In the hemolymph antibacterial protein group, most of the injured skin infected tissues formed a new transparent layer and cuticle, and there are a few new granulation tissues in the dermis. The newly formed hair follicles and sebaceous glands can be seen in the recovered skin area (Figure 3).

The number of inflammatory cells in the epidermis and dermis of the normal saline, erythromycin treatment, and *Z. morio* hemolymph treatment groups on the 3rd, 5th and 7th day was counted under the microscope. The results showed that the number of inflammatory cells in the normal saline group was significantly higher than that in the erythromycin and hemolymph treatment groups, which proved that *Z. morio* hemolymph can effectively reduce inflammatory cell infiltration (Supplementary Figure S4).

### 3.6. Z. morio Hemolymph Alleviates Inflammation of Local Thermally Injured Wound in Mice

**Figure 4:**
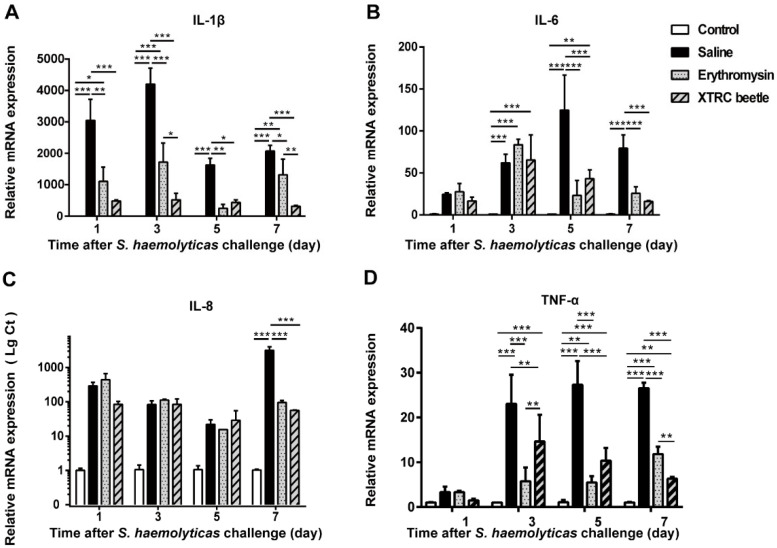
Effect of *Z. morio* hemolymph on the expression of IL-1β, IL-6, IL-8, and TNF-α after thermal injury and infection in mice. (**A**) Real-time fluorescent quantitative polymerase chain reaction (PCR) was used to detect the expression of IL-1β mRNA in the skin tissue of mice after thermal injury and infection. (**B**) Expression of IL-6 mRNA in skin tissue collected from mice after *S. haemolyticus* challenge was analyzed using quantitative real-time PCR. (**C**) Relative expression of chemokine IL-8 in normal control, saline, erythromycin ointment and *Z. morio* hemolymph groups. (**D**) Relative expression of TNF-α in wound skin tissue of thermally injured and infected mice after various treatments. All data are presented as mean ± standard deviation of the mean (SEM), * *p* < 0.05; ** *p* < 0.01; *** *p* < 0.001, *n* = 4.

IL-1β in the local tissue of mice in the saline group after thermal injury was significantly higher than that in the control group. After the treatment of *Z. morio* hemolymph antibacterial protein, the relative expression levels of IL-1β in mice were significantly lower than those in the saline group. Compared with the erythromycin group, the relative expression of IL-1β in *Z. morio* hemolymph antibacterial protein group decreased significantly on days 3 and 7 (Figure 4A). The relative expression levels of IL-6 in the hemolymph antibacterial protein treatment group were significantly different from those in the saline group on days 5 and 7 after thermal injury and infection (Figure 4B). The relative expression levels of IL-8 in the hemolymph antibacterial protein and erythromycin treatment groups on day 7 after thermal injury and infection were different from those in the normal saline group (Figure 4C). The expression levels of TNF-α after thermal injury and infection were significantly reduced in the hemolymph antibacterial protein treatment group and erythromycin groups (Figure 4D).

## 4. Discussion

Staphylococci can be transmitted in humans and animals [20]. Severe *Staphylococcus* skin wounds infections in humans and animals cannot be ignored. As one of the most promising substances to reduce the use of antimicrobials and bacterial drug resistance. AMPs have a strong killing effect on many microorganisms and can promote the wound healing process [21,22]. For patients with a diabetic skin ulcer, Kangfuxin Liquid, which is mainly composed of *Periplaneta americana* extract, combined with basic treatment, can significantly shorten the wound healing time of patients with skin ulcers [23]. AMPs kill bacteria by breaking the membrane or inhibiting intracellular function [24]. AMPs regulate inflammation by recruiting immune cells or initiating the immune system to promote bacterial clearance [25]. Unnatural amino-acid-based-star-shaped poly (l-ornithine)s can significantly reduce microbial load and improve thermal injury wound healing of *Pseudomonas aeruginosa*-infected mice [26]. Human cathelicidins LL-37 are associated with host stimulation events that are important for wound healing. LL-37 stimulates keratinocyte proliferation and induces angiogenesis [27,28]. In the mouse model, the synthetic β-folded peptide IK8L can potentially treat burn wounds infected by *Pseudomonas aeruginosa* in vivo [29].

In our previous study, a natural hemolymph antimicrobial extract from *Z. morio* had a positive antibacterial effect, which can maintain antibacterial ability after various physical and chemical treatments [17]. At the same time, it does not have hemolysis ability and obvious cytotoxicity at an effective bactericidal concentration range [17]. In this study, we successfully established a model of *S. haemolyticus* infection after thermal injury in mice skin. The main antibacterial components in *Z. morio* hemolymph were analyzed for the first time by preparative liquid chromatography and shotgun proteomics, including coleoptericin, coleoptericin B, defensin, isoform B and C, and attacin 1a. As a therapeutic drug, *Z. morio* hemolymph showed a positive therapeutic effect in the skin thermally injured infected by *S. haemolyticus.* It accelerated wound healing, reduced pathological damage of skin tissue and alleviated local inflammation.

In all patients with postburn infection, the symptoms of continuous elevated wound area temperature are accompanied [30]. Our results showed that the wound area temperature of mice increased significantly after thermal injury and infection and was higher than normal for 6 days. Both *Z. morio* hemolymph group and erythromycin groups could reduce the wound area temperature of mice on day 2 after infection. There was no significant difference compared with the control group. The wound area temperature of hemolymph antibacterial protein treatment group was closer to the control group, which suggests that hemolymph can relieve symptoms of elevated local wound temperature. *Z. morio* hemolymph showed excellent antibacterial activity in vitro, and had a superior bactericidal effect on *S. aureus* ATCC25923 [17]. Our study showed that the *Z. morio* hemolymph can significantly reduce the load of *S. haemolyticus* in the wound tissue of mice, which may be related to a previous report, in which *Z. morio* hemolymph can increase the permeability of the bacterial membrane, destroy cell membrane integrity and kill the bacteria [17]. White blood cell count was used for early diagnosis of postburn infection [31]. The number of leukocytes in the peripheral blood of mice increased significantly after infection. On day 5 of treatment, *Z. morio* hemolymph could significantly reduce the number of peripheral blood leukocytes in thermally injured and infected mice.

The wound healing of mice after thermal injury occurs mainly through wound contraction repair, which increases the healing time of mice [32]. Histological assessment, wound area, and re-epithelialization are important indicators for wound healing [33]. Therefore, measuring the recovery rate of the wound area after thermal injury in mice is one of the most important indicators of the thermal injury infection model in mice. Our results showed that the *Z. morio* hemolymph significantly reduced the wound area on day 5 after burn infection. At the same time, the experimental sites of the three groups of mice at different time points were sampled and sliced. Histopathological observation showed that the *Z. morio* hemolymph treatment group generated granulation tissue on day 5. The inflammatory reaction in the tissue was milder than that of the other two groups. Haidari et al, demonstrated that AgNP hydrogel treatment was helpful to reduce inflammation by reducing the number of neutrophils in the wound and increasing the proportion of anti-inflammatory M2 macrophages [34]. Therefore, we quantified the number of neutrophils in tissues, and the results showed that antibacterial hemolymph can reduce the number of inflammatory cells and relieve the inflammatory response.

Based on the above results, the erythromycin treatment group with a positive bactericidal effect does not have the same ideal therapeutic effect. Studies have proved that small molecular antimicrobial peptides have other regulatory effects. For example, Tiger17 (11-mer) induces macrophages to enter the site of inflammation through migration and proliferation of corneal cells, re-epidermalization and granulation tissue formation, promoting wound healing. It can also significantly increase the activation of c-Jun N-terminal kinase (JNK) and extracellular-signal-regulated kinase (Erk) sub-groups in mitogen-activated protein kinase (MAPK) signaling pathway, increase the release of transforming growth factor beta 1 (TGF-β1) and promote wound healing [35]. Although hemolymph has a good bactericidal effect, it can prevent the production of inflammatory factors in bovine mammary epithelial cells challenged by *Escherichia coli* to protect cells from pyroptosis [17]. The mechanism of some active ingredients in *Z. morio* hemolymph, except sterilization to promote wound healing, needs further study. 

Excessive edema and inflammation will delay wound healing [36]. When we collected tissues, we found severe subcutaneous edema in the saline group. Serum levels of pro-inflammatory cytokines, IL-1β, IL-6, and TNF-α increased after skin burns [37,38]. In this study, IL-1β, IL-6, and TNF-α in mice tissues were significantly increased at the early stage after thermal injury and infection, which was consistent with the results reported by Lateef et al [39]. Compared with the saline group, the *Z. morio* hemolymph treatment group can significantly reduce the expression of pro-inflammatory cytokines, suggesting that the *Z. morio* hemolymph could relieve the inflammatory response. IL-8 is the key medium for neutrophil recruitment during repair [40], and it is also an important factor in stimulating repair response [41]. Compared with the control group, the expression of IL-8 mRNA in the thermally injured mice group was increased. On day 7, there was a significant difference between the treatment and the saline groups, indicating that the repair response was fully activated. 

## 5. Conclusions

In this study, we first applied the antibacterial peptide *Z. morio* hemolymph to treat skin trauma in mice infected by *S. haemolyticus* and found a positive therapeutic effect, which was better than erythromycin ointment, a commonly used antimicrobial in clinics. *Z. morio* hemolymph can reduce the wound bacterial load, promote wound repair, and limit the inflammatory response, which helps in determining the application value of *Z. morio* hemolymph for antimicrobial therapy and provides basic data for the future clinical development of the effective treatment of skin thermal injury and bacterial infection.

## Figures and Tables

**Table 1:** Antibacterial protein component in *Z. morio* antimicrobial hemolymph.

Sequence Number	Description	Peptide Fragment Sequence
P80032	Coleoptericin	DHDFNAGWGK
P80033	Defensin, isoform B and C	FTCDVLGFEIAGTK
A0A076G362	Coleoptericin B	GQDHDFNAGWGK
A0A0345F0W3	Attacin 1a	SEPFFGGFVR

## Data Availability

The data presented in this study are available in the manuscript.

## Supplementary Materials

The following are available online at https://www.mdpi.com/article/10.3390/vetsci8120319/s1, Table S1: Real-time PCR Primers. Figure S1: Purification of *Z. morio* hemolymph by liquid chromatography. Figure S2: Analysis of main antimicrobial proteins in *Z. morio* hemolymph by shotgun proteomic. Figure S3: Trends of wound area temperature and body weight of mice in 11 days after thermal injury infection under different treatments. Figure S4: The number of inflammatory cells in the epidermis and dermis of normal saline, erythromycin treatment and hemolymph treatment groups on the 3rd, 5th and 7th day were counted under the microscope.
