# Supplementary material for: Therapeutic Effect of Darkling Beetle (Zophobas morio) Hemolymph on Skin Thermal Injury in Mice Infected by Staphylococcus haemolyticus
Source: Vet Sci. 2021 Dec 10;8(12):319. doi: 10.3390/vetsci8120319 (PMC8705897; doi:10.3390/vetsci8120319)
Supplement: Supplementary file 1 [file vetsci-08-00319-s001.zip › vetsci-1452729-supplementary.pdf]

Therapeutic Effect of Darkling Beetle (*Zophobas morio*) Hemolymph on Skin Thermal Injury in Mice Infected by *Staphylococcus haemolyticus*

Shu-Xian Li[a], Ning Liu[a], Meng-Ze Du[b*], Yao-Hong Zhu[a*]

[a] College of Veterinary Medicine, China Agricultural University, Beijing, China

[b] College of Animal Science and Technology, Beijing University of Agriculture, Beijing, China

* Corresponding author:

Mengze Du, College of Animal Science and Technology, Beijing University of Agriculture

No. 7 Beinong Road, Beijing 102206, P. R. China

Tel.: +86 010 8079 9149; Fax: +86 010 8079 9141 E-mail: dumengze@bua.edu.cn

Yaohong Zhu, College of Veterinary Medicine, China Agricultural University

No. 2 Yuanmingyuan West Road, Beijing 100193, P. R. China

Tel.: +86 010 6273 1094; Fax: +86 010 6273 1274 E-mail: zhuyaohong@cau.edu.cn–

1 Supplementary Tables

Table S1. Real-time PCR Primers.

Primers Name	Direction [a]	Sequence (5′→3′)	Accession Number
β-actin	F	GCTCTTTTCCAGCCTTCCTT	NM_007393.5
	R	GATGTCAACGTCACACTT	
IL1β	F	TGCCACCTTTTGACAGTGATG	NM_008361.4
	R	AAGGTCCACGGGAAAGACAC	
IL6	F	TTCCATCCAGTTGCCTTCTT	NM_001314054.1
	R	CAGAATTGCCATTGCACAAC	
IL8	F	CGGCAATGAAGCTTCTGTAT	NM_011339.2
	R	CCTTGAAACTCTTTGCCTCA	
TNF-α	F	ACGGCATGGATCTCAAAGAC	XM_006504297.5
	R	AGATAGCAAATCGGCTGACG	

[a] F = forward; R = reverse.

2 Supplementary Figures

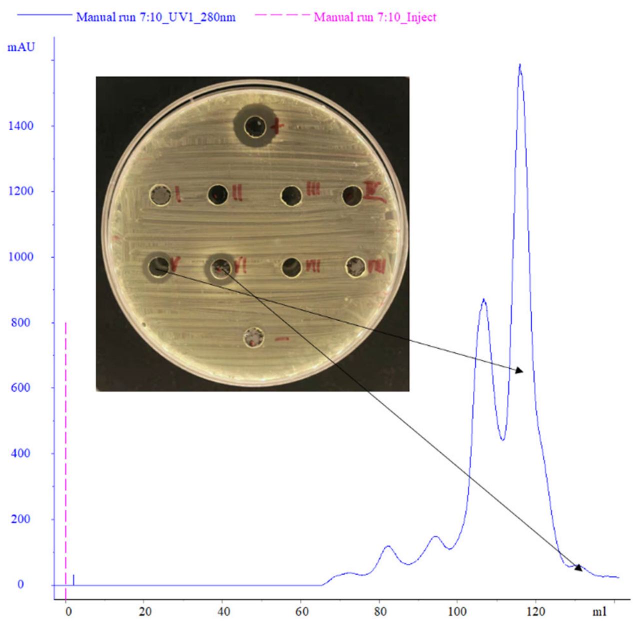

Figure S1. Purification of *Z. morio* hemolymph by liquid chromatography. Antibacterial protein was filtered by GE Superdex 100 pg chromatography column through liquid chromatography. According to the elution time, a total of 8 eluents were collected and 6 peaks were observed. Then we collected the eluents and freeze-dried it for in vitro antibacterial test. The eluents in tubes 5 and 6 contained antibacterial protein components.

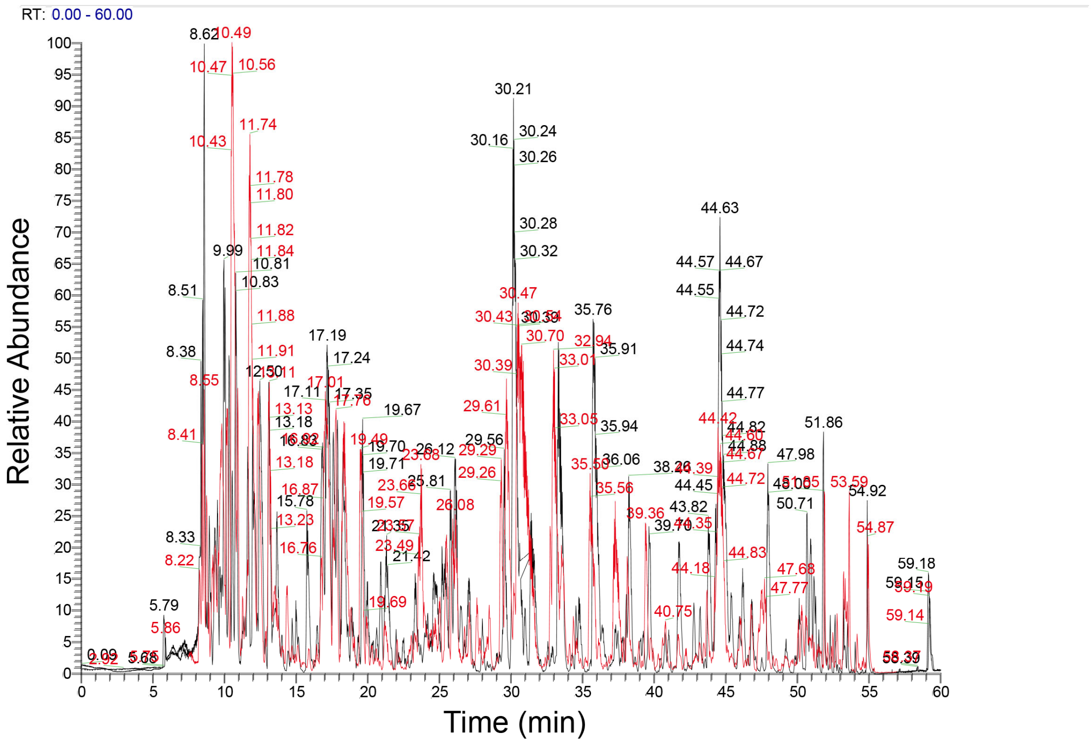

Figure S2. Analysis of main antimicrobial proteins in *Z. morio* hemolymph by shotgun proteomics. Red peak represents unimmunized *Z. morio* hemolymph protein samples, black peak represents molecular sieve preparation for liquid chromatography purification component samples.

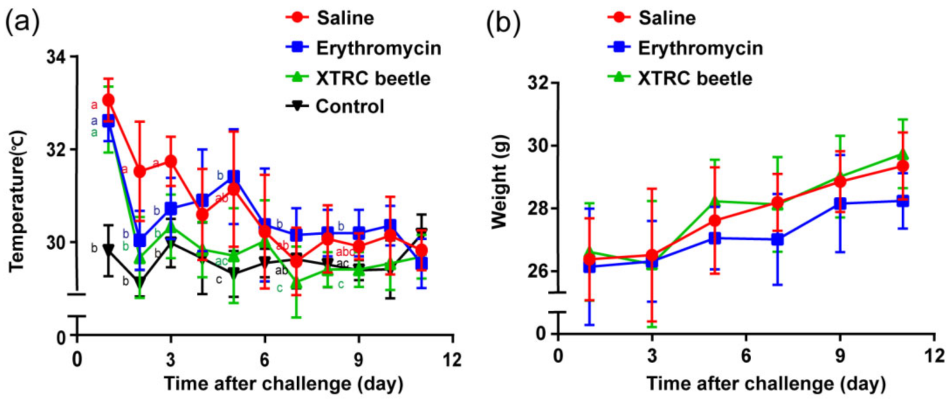

Figure S3. Trends of wound area temperature and body weight of mice in 11 days after thermal injury infection under different treatments. (a) Local body surface temperature of thermal injury infection wound in mice. (b) Body weight changes of mice in each group after thermal injury infection. Mean values at the same time point without a common superscript (a, b, c) differ significantly ($P < 0.05$; Tukey's test), and the data are expressed as the mean ± standard error of the mean (SEM), n=6.

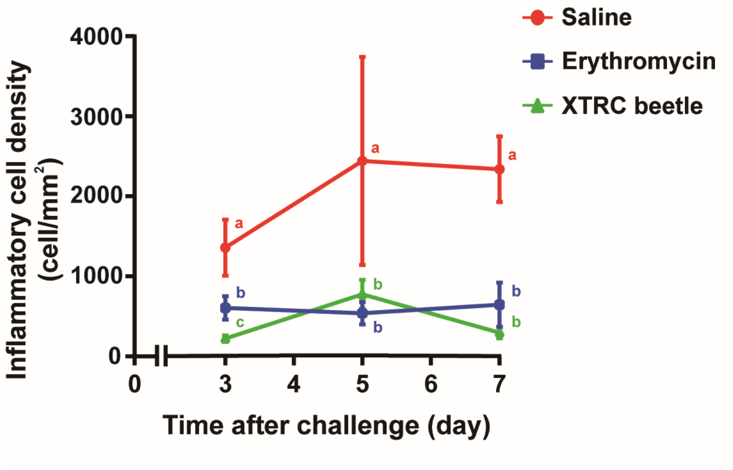

Figure S4. The number of inflammatory cells in the epidermis and dermis of normal saline, erythromycin treatment and hemolymph treatment groups on the 3rd, 5th and 7th day were counted under the microscope. Mean values at the same time point without a common superscript (a, b) differ significantly ($P < 0.05$; Tukey's test), and the data are expressed as the mean ± standard error of the mean (SEM), n=4.
